# Enzyme-based amperometric galactose biosensors: a review

**DOI:** 10.1007/s00604-017-2465-z

**Published:** 2017-08-25

**Authors:** Prosper Kanyong, Francis D. Krampa, Yaw Aniweh, Gordon A. Awandare

**Affiliations:** 1 West African Centre for Cell Biology of Infectious Pathogens (WACCBIP), University of Ghana, Legon, Accra, Ghana; 2 School of Engineering, Ulster University, Jordanstown, BT37 0QB UK; 3 Department of Biochemistry, Cell & Molecular Biology, University of Ghana, Legon, Accra, Ghana

**Keywords:** Galactose oxidase, Electrochemistry, Enzyme immobilization, Modified electrodes, Hydrogen peroxide, Glucose, Redox mediators, Screen-printed electrodes, Disposable sensors, Galactose dehydrogenase

## Abstract

This review (with 35 references) summarizes the various strategies used in biosensors for galactose, and their analytical performance. A brief comparison of the enzyme immobilization methods employed and the analytical performance characteristics of a range of galactose biosensors are first summarized in tabular form and then described in detail. Selected examples have been included to demonstrate the various applications of these biosensors to real samples. Following an introduction into the field that covers the significance of sensing galactose in various fields, the review covers biosensors based on the use of galactose oxidase, with a discussion of methods for their immobilization (via cross-linking, adsorption, covalent bonding and entrapment). This is followed by a short section on biosensors based on the use of galactose dehydrogenase. The conclusion section summarizes the state of the art and addresses current challenges.

**Graphical abstract:**
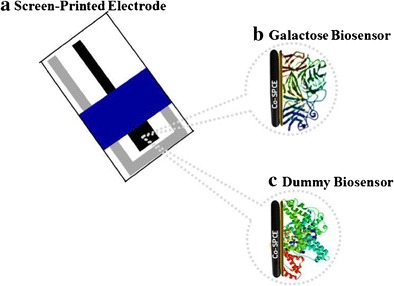
Fabrication of a disposable screen-printed (a) electrochemical galactose biosensor (b) for real sample analysis and a dummy biosensor (c) for compensating the effect of interferences

Fabrication of a disposable screen-printed (a) electrochemical galactose biosensor (b) for real sample analysis and a dummy biosensor (c) for compensating the effect of interferences

## Introduction

The quantitative determination of galactose is of great importance in clinical chemistry, food and fermentation industries. There are two general analytical methods for the analysis of galactose in real samples, namely, separation by liquid or gas chromatography, and enzyme-based methods using galactose oxidase (GalOx) or galactose dehydrogenase (GADH) in conjunction with spectrophotometric, polarimetric, and fluorometric detection of enzymatic products [1, 2]. Several commercial assay kits based on GADH have been developed. However, these methods are usually cumbersome and expensive, time-consuming and often require skilled personnel to operate them [1–3]. Electrochemical measurements, especially in conjunction with the use of biosensors, are most attractive for galactose analysis because they are rapid, relatively less expensive and sensitive and does not require pre-separation/treatment procedures [2, 3]. This review discusses amperometric biosensors constructed based on GalOx or GADH, the method of enzyme immobilization, and where applicable, the application of the sensors to the analysis of real samples. To the best of our knowledge, this is the first time galactose biosensors have been reviewed.

## Galactose oxidase-based biosensors

**Scheme 1:**
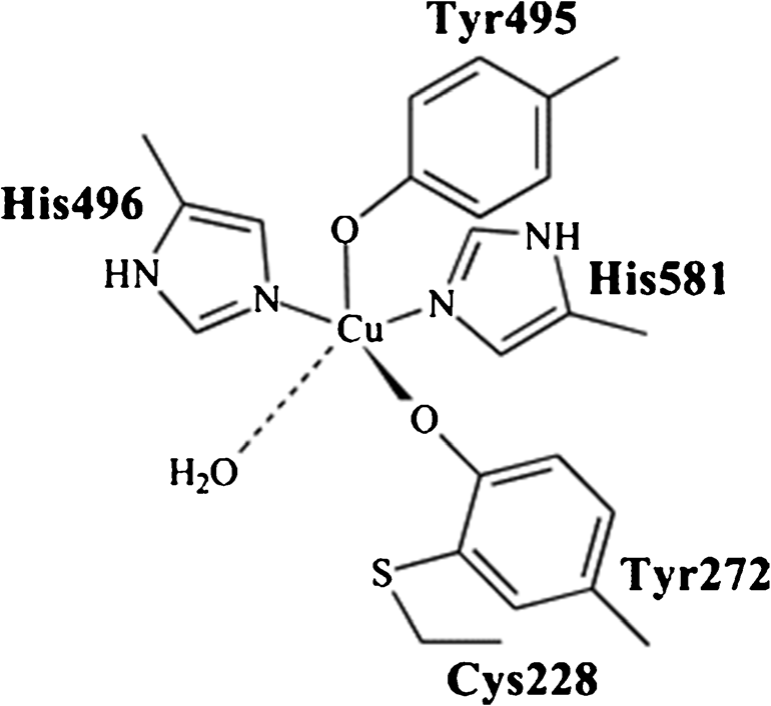
Active site of galactose oxidase showing coordinating ligands (Reprinted from [4] with permission from Elsevier)

Galactose oxidase (GalOx) is a free radical enzyme in which the active site contains five free radical ligands; namely two tyrosines, two histidines and a solvent molecule that is usually water. These free radicals are covalently linked to a copper center (Scheme 1) [4].

The electrochemical, biochemical and structural characteristics of GalOx has been extensively reviewed and discussed elsewhere [4, 5]. GalOx catalyzes the stereospecific oxidation of *D*-isomers of a range of primary alcohol substrates such as *D*-galactose, dihydroxyacetone (DHA) as well as polysaccharides with *D*-galactose at their reducing end [4, 5].

Good biosensor selectivity is based on the enzyme catalysis of a substrate; in this case GalOx can be utilized to catalyze galactose. The electrochemical response process can be described by the following equation (Eq. 1):

1 $$ \upalpha -\mathrm{D}-\mathrm{galactose}+{\mathrm{O}}_2\kern0.5em \underrightarrow{\mathrm{GalOx}}\kern1em \upalpha -\mathrm{D}-\mathrm{galactohexodialdose}+{\mathrm{H}}_2{\mathrm{O}}_2 $$

The electrochemical monitoring of the enzymatically produced H_2_O_2_ at an appropriate transducer generates the analytical response [2, 6]. The amperometric monitoring of H_2_O_2_ can be done either via its oxidation (anodic response) or reduction (cathodic response) at the surface of an electrode. Electrodes modified with metal nanoparticles, metal oxides, graphene and graphene oxides and ionic liquids have been employed during the analysis of H_2_O_2_ [6, 7]. The anodic and cathodic behavior of H_2_O_2_ can be described by Eqs. 2 and 3, respectively;

2 $$ {\mathrm{H}}_2{\mathrm{O}}_2\to {\mathrm{O}}_2+2{\mathrm{H}}^{+}+2{\mathrm{e}}^{-} $$

3 $$ {\mathrm{H}}_2{\mathrm{O}}_2+2{\mathrm{e}}^{-}+2{\mathrm{H}}^{+}\to 2{\mathrm{H}}_2\mathrm{O} $$

Typically, the use of redox mediators within the working electrode can allow H_2_O_2_ to be detected at low potentials [2, 3, 6]; thus, substantially reducing the effect of common interfering species in real samples. These favorable aspects of enzyme-based electrochemical biosensors would allow for the development of a rapid, selective and low-cost analytical devices; clearly, these are required for point-of-care applications.

### Cross-linking

The first electrochemical method for quantitative galactose determination in biological fluids was reported by Taylor and his co-workers in 1977. A rapid, precise and accurate galactose sensing micro-method was developed by incorporating GalOx into glutaraldehyde (Glu). The GalOx-Glu mixture was subsequently casted onto a polycarbonate membrane (PC) and allowed to air dry [8]. The GalOx-Glu-PC system was then used to modify the Model 23A Glucose Analyzer and employed for the analysis of galactose in both plasma and whole blood. Of the 39 compounds screened, the only physiologically relevant interference found was glycerone. The biosensor detected galactose up to 28.0 mM, however, the lower limit of detection was not stated. The detection of physiologically relevant species in complex matrices such as urine and whole blood using traditional electrode materials always present a challenging and significant problem with regards to the effect of interferences. In view of this, Manowitz and his co-workers [9] developed a biosensor that employed a composite polymer, with the ability to eliminate interferences. The system was used in conjunction with a flow injection analysis (FIA) system to detect galactose in human blood. To do this, a platinized carbon electrode was modified with a polymer film via the electropolymerization of 1,3-diaminobenzene/resorcinol monomers. Thereafter, the polymer modified-platinized carbon electrode was coated with 1.25% Glu followed by aliquots of GalOx solution. After allowing the electrode to air dry, a second layer of Glu was drop-casted to form a sandwich format sensor. The sensor showed improved selectivity to H_2_O_2_ and minimized the effect of common interferences such as ascorbic acid, urate and paracetamol. When the biosensor was employed for the analysis of galactose in human plasma, the linearity was found to be up to 6.0 mM with a lower limit of detection of 50.0 μM and a storage stability of well over 30 days at room temperature [9].

Wang et al., also utilized Glu as a linker for immobilizing GalOx onto the surface of a Pt disc electrode pre-coated with electrodeposited Chitosan/Prussian Blue (PB) thin films [10]. The final biosensor was then used to detect galactose in human blood serum and fermentation broth. The response time of the sensor to galactose was found to be in the range of 42–60 s with a sensitivity of 49.0 nA.mM^−1^. It was found to be stable up to 30 days at 25 °C with a precision for repeated measurements of 3.4%. The effect of interferences on the biosensor response was not evaluated. However, another study utilizing Glu as a cross-linker for immobilizing GalOx, based on the transduction capabilities of multi-walled carbon nanotubes (MWCNTs) and graphene composites, significantly eliminated the effect of interferences [11]. The first sensor was based on graphene, Co_3_O_4_ nanoparticles and chitosan composite modified glassy carbon electrode (GCE) while the second electrode was made from a composite of MWCNTs, Co_3_O_4_ nanoparticles and chitosan. In both cases, GalOx was immobilized onto the modified GCE surface by cross-linking with Glu. When the biosensors were applied to the analysis of galactose in human serum samples, the MWCNTs-based biosensor was found to have over 1.6-fold increase in sensitivity with a linearity range and detection limit far more superior to the graphene-based one. The electroanalytical performance of the two galactose biosensors are summarized in Table 1.

**Table 1:** Analytical performance characteristics of galactose oxidase-based amperometric biosensors

Sensor design	Sensitivity	LOD (μM)	Linear range (mM)	Response time (s)	Storage stability (days)/K_m_ (mM)	Real sample	Ref
GalOx/Ferrocene/Graphite	0.9 mA.M^−1^	2.8	5.6 × 10^−3^ – 0.6	44	52/−	-	[1]
GalOx/CA/Co-SPCE	7.0 μA.mM^−1^.cm^−2^	20.0	0.1–25.0	30	14/~12.5	Serum	[2]
GalOx/CA/Co-SPCE	3.1 μA.mM^−1^.cm^−2^	0.2	1.98–9.25	-	14/−	Culture medium	[3]
GalOx/Glu/PC/H_2_O_2_ electrode	-	-	0.0–28.0	40	7/−	Plasma, Blood	[8]
Glu/GalOx/Glu/1,3-DAB/Res/Pt	-	50.0	50 × 10^−3^ – 6.0	18	30/2.5	Plasma	[9]
GalOx/Glu/Chit/PB/Pt	49.0 nA.mM^−1^	60.0	0.1–6.0	42–60	30/−	Serum	[10]
GalOx/Co_3_O_4_/Graphene/GCE	6.6 μA.mM^−1^.cm^−2^	3.0	9.0 × 10^−3^ - 0.6	15	~30/1.2	Serum	[11]
GalOx/Co_3_O_4_/MWCNTs/GCE	10.4 μA.mM^−1^.cm^−2^	0.9	9.0 × 10^−3^ - 1.0	20	~30/0.7	Serum	[11]
GalOx/Nafion/Pt	100.0 nA.mM^−1^	-	0.25–4.25	<30	10/−	Serum	[13]
GalOx/PVF/O_2_ electrode	-	2.8 × 10^4^	28.0–168.0	-	90/−	Milk	[17]
GalOx/Collagen/ H_2_O_2_ electrode	1.0–3.0 mA.M^−1^	0.5	5.0 × 10^−4^ – 0.6	60	~300/−	Serum	[19]
PC/GalOx/Ferrocinium/CPE	-	-	-	<120	−/14.5	-	[21]
PU/GalOx/Polyacrylamide/O_2_/electrode	15.0–51.0 pA.mM^−1^	2.0	up to 1.5/10.0	0.5–5.0	30/−	-	[21]
GalOx/P3HT/SA/ITO	4.2 nA.mM^−1^	5.6 × 10^−3^	5.6–22.2	6	90/−	Milk	[22]
GalOx/PEG/Polyanion/Pt	106.0 nA.mM^−1^.cm^−2^	-	0.0–24.0	<40	−/43.0	-	[23]
GalOx/Glu/Chit/SWCNT-GCE	1126.0 nA.Mm^−1^	25.0	up to 1.0	-	−/−	Blood	[24]
GalOx/Poly-GMA-Co-VFc/Pt	23.0 nA.mM^−1^.cm^−2^	100.0	2.0–20.0	5	~30/16.0	-	[25]
GalOx/PEP/Au	1.8 μA.mM^−1^	25.0	2.0–16.0	5	~10/14.7	-	[26]
GalOx/Microtubules/ITO	~6.4 μA.mM^−1^.cm^−2^	10.0	0.1–1.0	30–40	7/−	-	[27]
GalOx/Polypyrrole/Pt	3.5–14.7 mA.M^−1^.cm^−2^	500.0	5.0 × 10^−4^ – 2.0	-	15/1.0–4.0	Blood	[28]
GalOx/Polypyrrole/Pt	37.5 nA.mM^−1^	-	up to 2.0	30	−/15.8	-	[29]
GalOx/PVF/Pt	~1.0 μA.mM^−1^	-	up to 40.0	30–40	3/21.7	-	[30]
GalOx/Polypyrrole-[p(HEMA)]/Pt	937.0 μA.M^−1^	25.0	5.0 × 10^−2^ – 10.0	70	270/−	Serum	[31]
GalOx/Laponite clay/Pt	85.0 mA.M^−1^.cm^−2^	1.0	1.0 × 10^−3^ - 1.6	5	~28/−	-	[33]
GADH/NADP^+^/Os/CPE	1.7 μA.mM^−1^.cm^−2^	2.0 × 10^2^	1.0–3.0	-	−/68	Milk	[35]

*Glu* glutaraldehyde, *PC* polymcarbonate membrane, *CPE* carbon paste electrode, *1,3-DAB* 1,3-diaminobenzene, *Res* resorcinol. *PU* polyurethane, *PVF* polyvinylferrocenium, *[p(HEMA)]* poly(2-hydroxyethyl methacrylate), *P3HT* poly-3-hexyl thiophene. *SA* stearic acid, *ITO* indium tin-oxide, *P-4-MP* Poly-4-methoxyphenol, *CPE* cabron paste electrode, *Os* osphendione, *GADH* galactose dehydrogenase, *PVF* polyvinyl formal

### Adsorption

Buffone et al., 1980 physically adsorbed GalOx from the fungus *Dactylium dendroides* onto the Model 23A electrode. The immobilized GalOx/electrode system was thoroughly evaluated and found to be suitable for monitoring the treatment of infants with galactosemia, because it required very small sample volumes (~25.0 μL of serum) and was completed within a few minutes. It also provided analytical recoveries below 5.6 mM [12]. However, the analytical performance characteristics such as sensitivity, linear range and detection limit was not stated. Ji and Zhang (1993) immobilized GalOx onto a platinized GCE pre-coated with Nafion [13]. The presence of Nafion, a permeation-selective membrane, in the sensor design helped reduce the effect of interfering electroactive species such as uric acid and ascorbic acid. The biosensor exhibited a linear range of 0.25–4.25 mM and a response time of 30 s. There were no obvious variations in the sensor response for 300 repeated measurements in 10 days. Miyata et al., 1995 constructed a micro-sensor by adsorbing GalOx onto a porous carbon electrode pre-coated with a tris(2,2′-bipyridine) complex of osmium (II/III) as a redox mediator and utilized it for the detection of galactose [14]. The amperometric response of the biosensor to galactose was found to be linear over concentrations ranging from 10.0 mM to 5.0 × 10^3^ mM, with a correlation coefficient of 0.999. However, these biosensors did not have prolonged stability.

In view of this, a galactose biosensor was fabricated by drop-coating GalOx on the surface of a graphite electrode adsorbed with the redox mediator, ferrocene from paraffin solution [1]. The storage stability of the biosensor was highly improved when the sensor was treated with both diethylaminoethyl (DEAE)-dextran and inositol. It exhibited a low limit of detection of 2.83 μM, a response time of 44.0 s and linearity range of 5.6–610.6 μM. During repeated assays for over 5 h, there was only 5% drop in the original response; thus, indicating that the biosensor was highly stable and suitable for routine analysis of galactose. Mannino, Cosio and Buratti, 1999 fabricated a sensitive, specific and rapid biosensor for measuring galactose content in dairy products [15]. The biosensing probe consisted of rhodium-on-carbon paste with adsorbed GalOx. The sensor exhibited enhanced selectivity (with discrimination against interferences such as ascorbic acid) and enzymatically produced H_2_O_2_ was measured at lower potentials. The biosensor response was linear from 1.4 mM to 22.2 mM with a limit of detection of 0.5 mM [15]; these analytical performance characteristics demonstrated that the rhodium-on-carbon paste/GalOx electrode was a robust amperometric biosensor that can be employed for quality control in the dairy industry.

Jia et al., 2003 utilized thin-film technology to fabricate a microchip sensor strip consisting of a Pt disk as the working electrode [16]. Firstly, the Pt disk was coated with Nafion thin-film followed by GalOx. The presence of Nafion within the sensor design significantly enhanced its selectivity towards galactose in serum samples. The response time for galactose was <40 s with a linearity range from 0.1 mM to 8.0 mM. The precision for repeated measurements was ~1.7% and the influence of common interferences such as ascorbic acid, uric acid and glucose did not cause any significant change to the analysis of galactose; this high selectivity for galactose was attributed to the ability of the Nafion membrane to remove interferences. To validate the sensor, it was employed to recover known concentrations of galactose spiked into serum samples and the recovery values ranged from 97.0% to 105.0%; this demonstrated the suitability of the biosensor for routine analysis of galactose in real samples. Lastly, the biosensor retained 90.0% of its initial activity towards galactose following 1 month of storage. However, there was a 3-fold increase in the stability of a similar biosensor developed by adsorbing GalOx onto polyvinyl fluoride (PVF) membrane to form GalOx/PVF system. The GalOx/PVF layer was then attached to an O_2_ electrode and used for the determination of galactose in milk and milk products [17]. The response of the bioelectrode to standard concentrations of galactose was found to be linear from 28.0 mM to 168.0 mM (the former being the limit of detection). However, the biosensor did not eliminate the effect of interferences during the analysis of real samples.

**Fig. 1:**
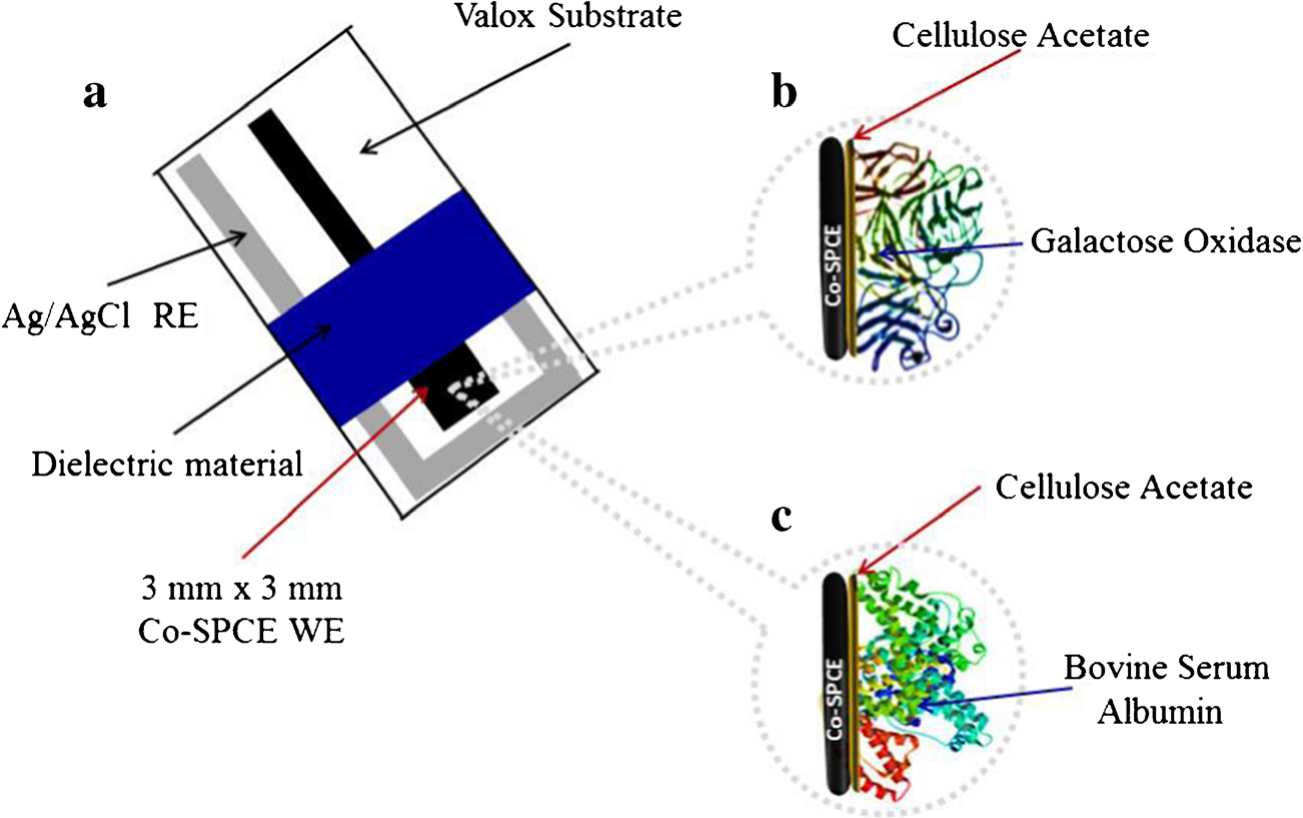
Design of **a** cobalt phthalocyanine screen-printed carbon electrode (Co-SPCE); **b** galactose biosensor and; **c** Dummy biosensor. RE: Reference Electrode, WE: Working Electrode

In another study [18], GalOx was physically adsorbed onto microporous polyacrylonitrile thin films. The biosensor appeared to have good stability and was highly reproducible. When the sensor was employed for the determination of galactose in human sera, the amperometric current responses increased linearly with increasing galactose concentrations from 20.0 μM to 1.6 mM. Kanyong et al., 2013 developed a disposable amperometric biosensor (Fig. 1a) for the measurement of circulating galactose in infant serum [2]. The sensor designed comprised of a water-based screen-printed carbon electrode (SPCE), incorporating the redox mediator, cobalt phthalocyanine (CoPc) (Co-SPCE). Firstly, Co-SPCE was covered with cellulose acetate (CA) followed by physical adsorption of GalOx (Fig. 1b). The operation of the biosensor was based on the interaction of galactose with the immobilized GalOx which leads to the production of H_2_O_2_; the H_2_O_2_ then diffuses through the pores of the CA membrane onto the underlying electrode where it undergoes a chemical oxidation by the CoPc (Co^2+^) to produce CoPc (Co^+^) (Eq. 4):

4 $$ 2{\mathrm{Co}}^{2+}{\mathrm{H}}_2{\mathrm{O}}_2\to 2{\mathrm{Co}}^{+}+{\mathrm{O}}_2+2{\mathrm{H}}^{+} $$

The electrochemical re-oxidation of Co^+^ to Co^2+^ (Eq. 5) occurred at an applied voltage of +0.5 V to produce the analytical response:

5 $$ {\mathrm{Co}}^{+}\to {\mathrm{Co}}^{2+}+{e}^{-} $$

Clearly, the detection of galactose via this system has the advantage that the H_2_O_2_ will pass through the small pores of the CA membrane that would exclude interferences commonly present in human bodily fluids.

The effect of temperature, ionic buffer strength, enzyme loading and pH on the analytical performance characteristics of the biosensor was thoroughly optimized. To compensate for the influence of interferences in serum that was not eliminated via the use of the CA membrane, the researchers fabricated a dummy biosensor (Fig. 1c) by using the same mass of bovine serum albumin (BSA) as that of the enzyme. The effects of interferences from the serum were measured with the dummy biosensor and subtracted from that of the galactose biosensor response. Using the dummy biosensor in conjunction with the galactose biosensor, the influence of several interfering species such as acetaminophen, uric acid, urea and ascorbic acid was successfully compensated for. The amperometric response of the optimized galactose biosensor on exposure to standard concentrations of galactose was linear from 0.1 mM to 25.0 mM with a sensitivity of 7.0 μA.mM^−1^.cm^−2^ and a calculated limit of detection of 20.0 μM. When the biosensor was used to analyze serum samples, the precision obtained on unspiked and spiked serum (*n* = 6) were 1.10% and 0.11%, respectively with a recovery of 99.9%.

Using the same biosensor design, the researchers fabricated a microband galactose biosensor for real-time monitoring of the update of galactose by hepatocellular carcinoma cells after exposing the cells to various toxic compounds [3]. The biosensor maintained steady-state current responses in culture medium for batch-type analysis to monitor the depletion of galactose by hepatocytes in a cell density-dependent manner. It provided a sensitivity of 7.3 μA.mM^−1^.cm^−2^, a linear range up to 9.52 mM and a coefficient of variation of 1.2%.

### Covalent bonding

A selective biosensor for galactose analysis was developed through covalent attachment of GalOx onto highly-polymerized collagen films [19]. An activated collagen film was immersed in GalOx solution at alkaline pH; thus, the enzyme was covalently linked to the collagen by reaction with acyl azides. The enzyme/collagen film system was then attached to a Pt anode, whose potential was maintained at +0.65 mV vs. Ag/AgCl reference electrode, and used for monitoring the enzyme-generated H_2_O_2_. To compensate for electrochemical interferences in biological fluids, a dummy electrode was used to record the interfering current. The response of the dummy electrode was then subtracted from the response of the enzyme-based electrode; thus, providing an accurate measurement of the enzymatically generated H_2_O_2_. A steady-state response was obtained within 5 min and the biosensor exhibited a calibration linearity from 0.5 μM to 600.0 μM and depending on the enzyme membrane activity, a sensitivity in the range of 1.0–3.0 mA.M^−1^ was found.

Galactose is an important substrate for fermentation and food production. Lundback and Olsson reported an amperometric technique for the detection of galactose in such systems by immobilizing GalOx onto reactors [20]. The enzyme was immobilized via aryloamino-derivatized controlled-pore glass and packed into the reactors. The H_2_O_2_ detection was mediated via hexacyanoferrate (II). The response of the sensor to standard solutions of galactose was found to be linear from 2.0 μM to 60.0 μM with a throughput of 45 samples/h and a relative standard deviation of 0.4%. In another study, Peteu, Emerson and Worden (1996) covalently attached GalOx onto the tip of a Clark-type O_2_ microelectrode [21]. The enzyme was mixed with a homogenous solution of polyacrylamide and the tip of the electrode was held in contact with the solution for a few seconds and then withdrawn; this procedure was repeated several times until a small bulb (~40.0 μm) was formed at the tip of the electrode. The bulb was then allowed to air-dry. Using the same procedure, the tip of the electrode was coated with polyurethane membrane. The limit of detection of the sensor was found to be 2.0 μM with a linear range that is dependent on the thickness of the polyurethane film but extended up to 10.0 mM. It also exhibited response times of 0.5–5.0 s, a sensitivity of 51.0 × 10^−5^ mA.M^−1^ and a storage stability of up to 1 month [21].

**Fig. 2:**
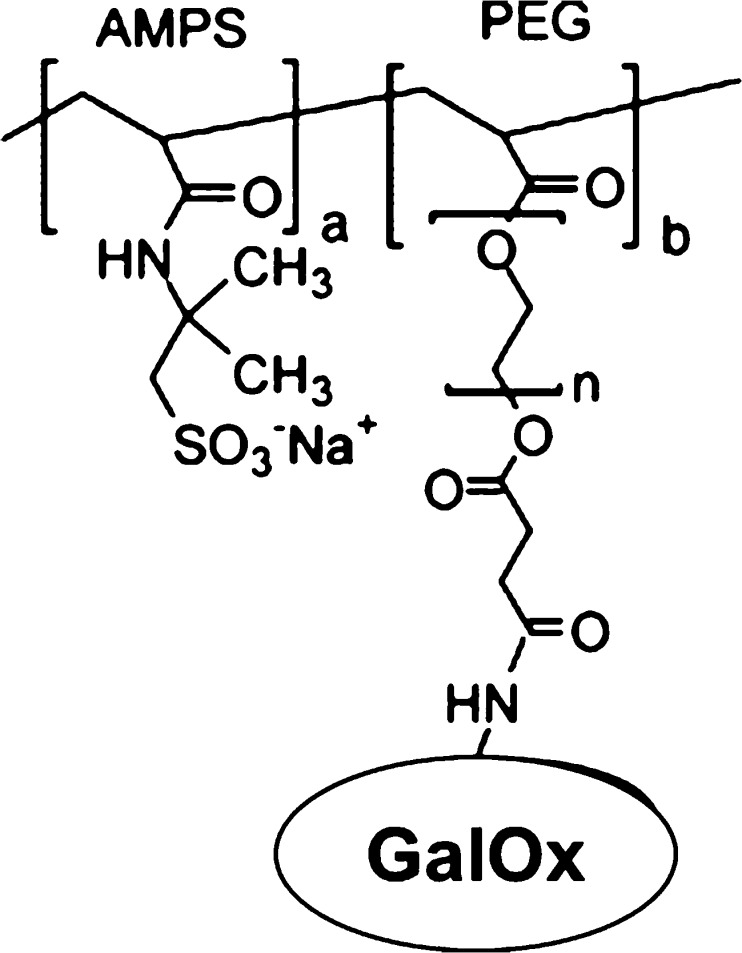
Schematic representation of GalOx immobilization to form a polyanion/PEG/GalOx conjugate. AMPS; 2-Acrylamido-2-methylpropane sulfonic acid (Polyanion) (Reprinted from [23] with permission from Elsevier)

Sharma et al., 2004 prepared an enzymatic amperometric biosensor for the determination of galactose in milk and milk products by immobilizing GalOx with poly-3-hexyl thiophene and with stearic acid onto an indium tin-oxide (ITO)-coated glass plate via Langmuir-Bloggett (LB) film deposition technique [22]. This design improved the shelf-life of the biosensor to well over 90 days at 40 °C storage conditions. The response time was 60 s with a linearity of 5.6–22.2 mM. In another study, galactose biosensors were fabricated by covalently attaching GalOx onto polyanion via polyethylene glycol (PEG) spacer (Fig. 2) [23].

The polyanion/PEG/GalOx conjugate was then entrapped within a polypyrrole film. This was done by electrodepositing onto a Pt electrode from a solution containing pyrrole and the polyanion/PEG/GalOx conjugated dopant; thus, the resulting polyanion/PEG/GalOx conjugate was effectively entrapped into the polypyrrole matrix. The amperometric response of the polypyrrole/polyanion/PEG/GalOx electrode to standard concentrations of galactose was found to be linear from 0.0 mM to 24.0 mM with a sensitivity of 106.0 nA.mM^-1.^cm^−2^ and an improved response time of ~40 s.

**Fig. 3:**
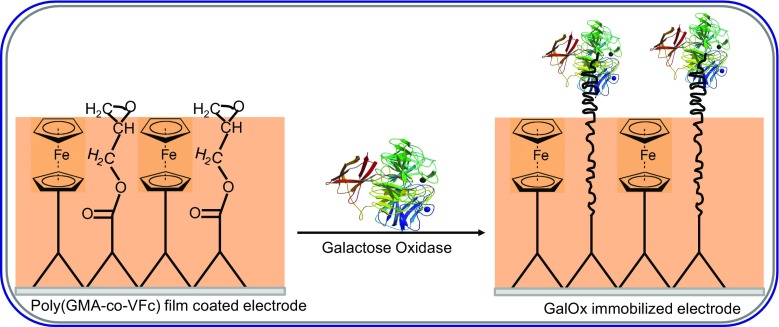
Structure of polyglycidyl methacrylate-co-vinyl ferrocene (Poly-GMA-co-VFc) and covalent binding of GalOx to the Poly-GMA- co-VFc film electrode (Redrawn from [25])

Tkac et al., utilized chitosan, a natural polymer, to solubilize carbon nanotubes. Further stabilization of the chitosan/carbon nanotube dispersion was done via chemical cross-linking with Glu. The free aldehyde groups of Glu were subsequently used to covalently immobilized GalOx [24]. To do this, a polished GCE was coated with the chitosan/carbon nanotube dispersion, followed by layers of Nafion, GalOx and Nafion, respectively. The use of the permeation-selective membrane, Nafion, to sandwich GalOx led to an interference-free detection of galactose in the presence of high concentrations of uric acid and paracetamol. The biosensor was found to be robust, reliable and was successfully used to analysis galactose in blood plasma with sample throughput of 150/h. The limit of detection was found to be 2.0 μM. When the biosensor was used in conjunction with amperometry and a Flow Injection Analysis (FIA) system to recover known concentrations of galactose spiked into blood plasma, the recovery index was found to be between 101.2 and 102.7%, with a precision of 2.0%. Another galactose biosensor was developed by covalently immobilizing GalOx onto a polymeric mediator, polyglycidyl methacrylate-co-vinyl ferrocene film via its amine groups (Fig. 3) [25].

Due the incorporation of the redox mediator, vinyl ferrocene, into the sensor design, H_2_O_2_ was measured at lower potentials and the final biosensor was found to have good stability, repeatability and reproducibility. The optimized sensor exhibited a linear response range of 2.0–20.0 mM, a limit of detection of 0.1 mM and a response time of 5 s. Similarly, a novel amperometric galactose biosensor was developed by immobilizing GalOx onto electrochemically synthesized poly(N-glycidylpyrrole-co-pyrrole) film via a one-step covalent attachment procedure and without the use of any coupling agents [26]. When the amperometric response of the biosensor was measured as a function of increasing concentrations of galactose, at an applied voltage of +0.7 V vs Ag/AgCl reference electrode, the response time was less than 5 s. The linearity range was from 2.0 mM to 16.0 mM with a sensitivity of 1.75 μA.mM^−1^. To enhance the sensitivity of galactose sensing, Lee et al., 2011 covalently attached galactose oxidase onto a bundle of conducting polymer microtubules [27]. The microtubules, prepared via the electropolymerization of 3,4-ethylenedioxythiophene (EDOT) onto a template, had structures which provided larger enzyme encapsulating space per unit area of the sensing electrode compared to other immobilizing procedures. When the biosensor was employed for the analysis of galactose, it exhibited a linear behavior from 0.1 mM up to 1.0 mM galactose with a sensitivity of 6.37 μA.mM^−1^.cm^−2^, a limit of detection of 10.0 μM (based on 3× the baseline noise) and a response time of 30–40 s.

### Entrapment

This method involves integrating an enzyme within the lattice of an appropriate polymer matrix or membrane. In addition to the immobilization of enzymes, membranes can also eliminate the effect of interfering electroactive species that may be present in real samples such as blood or urine [2, 3]. A series of amphiphilic pyrrolyl-alkylammonium ions differing in the size of their ammonium heads were used as a matrix for the immobilization of GalOx onto Pt and carbon disk electrodes [28]. Polypyrrolic films were electrogenerated from adsorbed amphiphilic pyrrole-galactose oxidase mixtures. The enzyme retention properties of the polymers were investigated and it showed that the lesser the hydrophobicity of the film, the higher the enzyme immobilization efficiency and the sensitivity of the biosensor was related to the permeability of the polymer. Compared to classical electrochemical enzyme entrapments, the method offered the possibility of controlling the composition of the enzyme-polymer film and allows for the determination of the amount of the immobilized enzyme; thus, its specific activity. There was no decrease in the enzymatic activity of the biosensor for up to 15 days and sensitivity values in the range of 3.5–14.7 mA.M^−1^.cm^−2^ were found for the various polymers with entrapped GalOx. Even though the influence of interfering species such as uric acid, ascorbic acid and paracetamol were found to decrease markedly with increasing polymer hydrophobicity, the sensor design was unable to eliminate the effect of the interferences.

In addition to this, a bio-platinum electrode for galactose was developed via the electrochemical polymerization of pyrrole in a buffer solution containing GalOx, which became entrapped in the growing polymer film during the electropolymerization [29]. The experimental conditions for this study ensured that the particles of polypyrrole were arranged tightly during the formation of the film; the space between the particles were sufficiently larger than the diameter of the enzyme; thus, the enzyme was entrapped in the polymer film based on the doping principles of conducting polymers. A Raman spectrum of the polypyrrole GalOx biosensor showed a characteristic peak for an amino acid at 1414 cm^−1^; an evidence for successful entrapment of the GalOx within the polypyrrole film. When the bioelectrode was used to analyze galactose, its response current increased linearly with increasing concentration of galactose up to 2.0 mM [29].

Gülce and co-workers [30] reported a Pt electrode coated with a redox polymer, polyvinylferrocenium (PVF^+^), which catalyzed the oxidation and reduction of organic species as well as H_2_O_2_. The chemical oxidation of H_2_O_2_ by the redox polymer, PVF+ occurred as (Eq. 6):

6 $$ 2{\mathrm{PVF}}^{+}{\mathrm{H}}_2{\mathrm{O}}_2\to 2\mathrm{PVF}+{\mathrm{O}}_2+2{\mathrm{H}}^{+} $$

followed by the electro-regeneration of the PVF at an applied potential of +0.7 V vs. SCE (Eq. 7):

7 $$ 2\mathrm{PVF}\to 2{\mathrm{PVF}}^{+}+2{e}^{-} $$

The matrix was used as a pre-concentration agent for the detection of inorganic anions. Consequently, the anion exchange properties of the redox polymer were utilized for developing the bioelectrode for galactose by immobilizing GalOx onto the matrix. When the bioelectrode was used to analyze galactose, a response time of 30–40 s was found and the upper limit of the linear working portion was 40.0 mM; these performance characteristics were deemed to be satisfactory for the analysis of galactose in bodily fluids.

Brahim et al., 2002 developed a rapid, two-step protocol for constructing galactose biosensors by entrapping GalOx within a polymeric composite [31]. The polymeric composite was formed as an interpenetration network of polypyrrole grown within a UV cross-linked-poly-(2-hydroxyethyl methacrylate) hydrogel. The sensor exhibited a linear response range, calculated limit of detection and response time of 5.0 × 10^1^–1.0 × 10^4^ μM, 25.0 μM and 70 s, respectively. When the biosensor was employed with amperometry for the analysis of serum samples, the precision values obtained for same day and day-to-day analyses were 3.8% and 4.4%, respectively, with recoveries ranging from 97.0% to 105.0%. Moreover, the biosensor eliminated the influence of interferences such as urate, paracetamol and ascorbate and retained 70.0% of its initial activity after 9 months of storage in a desiccator at 4 °C. Another polymer based-galactose oxidase sensor was fabricated via a one-step procedure via the electropolymerization of 4-methoxyphenol in the presence of GalOx onto a Pt electrode [32]. Polymeric thin films of 1.5 μm thickness with GalOx entrapped were prepared and the biosensor had a linear response to increasing concentrations of galactose up to 16.0 mM with a response time of <4 s. However, the sensor design did not eliminate the effect of interferences such as ascorbic acid, paracetamol and oxalic acid. The biosensor also lost well over 52% of its initial response to galactose within 30 days; thus, making it unsuitable for routine analysis of galactose.

An amperometric galactose biosensor was developed by entrapping GalOx within a laponite clay film coated onto a Pt electrode surface [33]. The laponite was found to provide a favorable environment for GalOx activity; consequently, the optimized sensor was found to respond linearly to increasing galactose concentrations from 1.0 μM to 1.6 mM (the former being detection limit) with a sensitivity of ~85.0 mA.M^−1^.cm^−2^. It was stable for up to 1 month at 4 °C and the response time was ~5 s. The effect of interferences on the biosensor response was not eliminated. In a similar study, GalOx was immobilized within the laponite clay matrix and the laponite-GalOx conjugate was deposited on screen-printed electrodes already modified with Prussian blue and poly-(*O*-phenylenediamine) [34]. Using the bioelectrode, the reduction of enzymatically generated H_2_O_2_ was performed at a significantly lower potential (−0.2 V vs Ag/AgCl) and the limit of detection for galactose was found to be 13.0 μM with a sensitivity of 16.0 mA.M^−1^.cm^−2^. Consequently, the laponite is deemed to be a suitable material for entrapping GalOx.

## Galactose dehydrogenase-based biosensors

The determination of galactose in real samples has been traditionally achieved via the use of the enzyme galactose oxidase. However, Maestre et al., 2005 utilized a galactose dehydrogenase (GADH) from thermoacidophilic archaebacterium [35]. GADH is a NADP^+^/NADPH dependent dehydrogenase having catalytic activity not only for galactose but also for other sugars such as glucose and xylose. In this study, pre-treated graphite powder, osphendione (Os) (a redox mediator), NADP^+^, GADH and paraffin oil were thoroughly mixed and packed into a Teflon tube to form a carbon paste electrode. The catalytic and electrochemical reactions for galactose based on GADH can be described by Eqs. 8–10:

8 $$ \upalpha -\mathrm{D}-\mathrm{galacto}\mathrm{se}+{\mathrm{NADP}}^{+}\kern0.5em \underrightarrow{\mathrm{GADH}}\kern1em \upalpha -\mathrm{D}-\mathrm{galacto}-1,5-\mathrm{lactone}+\mathrm{NADPH} $$

9 $$ 2\mathrm{NAPDH}+\mathrm{OsQ}\to 2{\mathrm{NADP}}^{+}+{\mathrm{OsQH}}_2 $$

10 $$ {\mathrm{OsQH}}_2\to \mathrm{OsQ}+2{\mathrm{H}}^{+}+2{\mathrm{e}}^{-} $$

When the biosensor was used with a FIA system to analyze galactose, the linear range was found to be from 1.0 mM to 3.0 mM with a limit of detection of 0.2 mM and precision values of 12.0–15.0%; these analytical performance characteristics indicated that the GADH catalysis of galactose using osphendione as a redox mediator was successful.

## Conclusion

This review has highlighted some of the novel approaches that have been employed for fabricating amperometric galactose biosensors. The analytical performance characteristics of these galactose biosensors are summarized in Table 1.

The use of GalOx offers an additional advantage over galactose dehydrogenase (GADH), as the latter requires co-factors to be co-immobilized onto the underlying transducer. In addition to this, the response times are generally shorter for GalOx based biosensors. Consequently, only one paper reported the use of GADH in its design. However, there are several drawbacks associated with the use of GalOx-based biosensors. Significantly higher applied potentials must be used in order to generate an electrochemical response from the enzyme generated H_2_O_2_.

Expensive electrode materials such as graphene, gold, platinum and carbon nanotubes are often used to develop galactose biosensors employing GalOx. However, three sensors employing GalOx immobilized onto screen-printed carbon electrodes (SPCEs) as the electrode material have been described. SPCEs offer an inexpensive method for fabricating galactose biosensors, which is crucial in the commercialization of such devices for point-of-care applications. Finally, there is a plenitude of GalOx from different sources; thus, making it relatively less expensive. Cost is a relevant point to be considered for commercialization of these devices.
